# Supplementary material for: Comparative multiomics analysis of cell physiological state after culture in a basket bioreactor
Source: Sci Rep. 2022 Nov 23;12:20161. doi: 10.1038/s41598-022-24687-4 (PMC9686226; doi:10.1038/s41598-022-24687-4)
Supplement: Supplementary file 1 — Supplementary Information 1. [file 41598_2022_24687_MOESM1_ESM.zip › raw data/Proteomic raw data/6.KEGGenrichment/EG.vs.CG/UP/pathway/src/04360.html]

map04360


- K04352

- Up regulated proteins

A0A0D9RQM0(Description:Uncharacterized protein;Gene:RASA1;log2FC:1.0151)

- K04352

- Up regulated proteins

A0A0D9RQM0(Description:Uncharacterized protein;Gene:RASA1;log2FC:1.0151)

Close
